# Role of Health Literacy in Health-Related Information-Seeking Behavior Online: Cross-sectional Study

**DOI:** 10.2196/14088

**Published:** 2021-01-27

**Authors:** Hee Yun Lee, Seok Won Jin, Carrie Henning-Smith, Jongwook Lee, Jaegoo Lee

**Affiliations:** 1 School of Social Work The University of Alabama Tuscaloosa, AL United States; 2 School of Social Work University of Memphis Memphis, TN United States; 3 School of Public Health University of Minnesota Minneapolis, MN United States; 4 Department of Global Health and Population Harvard T H Chan School of Public Health Harvard University Boston, MA United States; 5 School of Social Work Jackson State University Jackson, MS United States

**Keywords:** digital divide, health literacy, internet, technology, access

## Abstract

**Background:**

The internet has emerged as a main venue of health information delivery and health-related activities. However, few studies have examined how health literacy determines online health-related behavior.

**Objective:**

The aim of this study was to investigate the current level of health-related information-seeking using the internet and how health literacy, access to technology, and sociodemographic characteristics impact health-related information-seeking behavior.

**Methods:**

We conducted a cross-sectional study through a survey with Minnesotan adults (N=614) to examine their health literacy, access to technology, and health-related information-seeking internet use. We used multivariate regression analysis to assess the relationship between health-related information-seeking on the internet and health literacy and access to technology, controlling for sociodemographic characteristics.

**Results:**

Better health literacy (β=.35, SE 0.12) and greater access to technological devices (eg, mobile phone and computer or tablet PC; β=.06, SE 0.19) were both associated with more health-related information-seeking behavior on the internet after adjusting for all other sociodemographic characteristics. Possession of a graduate degree (β=.28, SE 0.07), female gender (β=.15, SE 0.05), poor health (β=.22, SE 0.06), participation in social groups (β=.13, SE 0.05), and having an annual health exam (β=.35, SE 0.12) were all associated with online health-related information-seeking.

**Conclusions:**

Our findings indicate that access to online health-related information is not uniformly distributed throughout the population, which may exacerbate disparities in health and health care. Research, policy, and practice attention are needed to address the disparities in access to health information as well as to ensure the quality of the information and improve health literacy.

## Introduction

Health information access and use are essential for optimal health outcomes [1]. For example, a meta-analysis found that health information access and use were associated with better compliance with medical treatment in patients with chronic and acute illnesses [2]. Another study demonstrated that health information access and use are associated with self-care behavior among patients with heart failure [3]. Furthermore, several previous studies revealed that health information access and use predict other health-related behaviors, including having an annual medical checkup and undergoing cancer screenings, because increased cognitive knowledge in health can lead to behavioral outcomes such as receiving preventive screenings [4-6]. This association shows that securing adequate access to and use of health information plays a key role in improving health outcomes in varied domains.

Since its advent, the internet has served as a primary medium to convey health information [7,8]. For the last few decades, the dramatic increase in the usage of internet-embedded devices such as laptops, tablets, and smartphones has enabled people to access and use health information at any place and time [9]. According to recent national surveys [10,11], in the United States, approximately three-quarters of adults have broadband internet service at home, nearly 90% use the internet, nearly two-thirds of adults own a smartphone, and nearly 80% use the internet for various health-related purposes such as seeking health information, communicating with doctors, and purchasing medicine or vitamins [12-14].

Although the internet has contributed to enhancing the potential for health information access and use while reducing barriers to obtaining health information in the US general population, not all people have obtained these benefits. For example, gender is a key predictor of use of the internet and online health information behavior [15]. Previous studies have shown that men are less likely than women to use the internet for health information–seeking and to trust online sources [13,16-18]. There remain some groups of people who still experience difficulties in accessing and utilizing health information because of barriers such as a digital gap and limited health literacy [1,19,20].

A digital gap is defined as unequal access to technology and capability of its usage [21]. These variations in access to and use of technology often lead to disparities in health outcomes [22]. Existing research shows that predictors of the digital gap include age, gender, education attainment, and income [20]. Closing the digital gap is important because it is significantly associated with the enhancement of health-related decision making, health behavior, and health care system navigation [23,24]. In addition to the digital gap, health literacy also influences health information access and use. Health literacy is defined as “the degree to which individuals can obtain, process, understand, and communicate about health-related information needed to make informed health decisions” [25]. Researchers agree on the importance of health literacy in that people with low health literacy are at a high risk of varied poor health outcomes [26-29].

Despite consistent emphasis on the importance of health literacy as a determinant of health outcomes [30,31], and the emergence of the internet as a main venue of health information delivery and health-related activities, few studies have examined how health literacy determines online health-related behaviors such as the usage of the internet for health information–seeking and health-related activities, including online scheduling for visits to clinics. Therefore, this study had a threefold purpose: (1) to investigate the levels of online health-related behavior, (2) to assess whether health literacy is associated with online health-related behavior, and (3) to examine whether sociodemographic characteristics are associated with online health-related behavior. The findings of this study will offer insights into health literacy and sociodemographic-specific interventions for improving online health-related behaviors and health outcomes.

## Methods

### Research Design and Data Collection

The research team collected survey data from 732 adults aged 18 years or older at the 2016 Minnesota State Fair with approval from the University of Minnesota Institutional Review Board. The survey included questions about health literacy and health behavior in addition to general sociodemographic information. Participants received a small gift (a backpack with the University of Minnesota logo, which is worth about US $3) after they completed their voluntary survey using REDCap software via an iPad. Owing to missing values, data from 614 respondents (241 men and 373 women) out of the original 732 were used in the study as the analytic sample. Comparing the included analytic sample of 614/732 (83.9%) to the excluded sample of 118/732 (16.1%) due to missing values, we found that people in the included sample, who completed the survey, were more likely to have a family cancer history (included sample: 480/614, 78.2%; excluded sample: 49/76, 64.5%; *P*=.02), have both a smartphone and a computer or tablet PC (included sample: 485/614, 79.0%; excluded sample: 36/63, 57.1%; *P*<.001), and use internet for health information (included sample: 549/614, 89.4%; excluded sample: 57/74, 77.0%; *P*=.02) at the 5% significance level.

### Instruments and Measures

To measure health-related information-seeking behavior, the main outcome variables of this study were based on two sets of questions about seeking health-related information on the internet. First, a survey question asked whether the respondent used the internet for health information, which was used as a binary outcome variable for health-related internet use. Second, the total score of the 12 health-related internet use items from the Health Information National Trends Survey [32] questionnaire was used to measure the participants’ health-related information-seeking internet use score. Each question was assigned a value of 0 (no) or 1 (yes). The Cronbach α of the 12 items for the health-related internet use score was .71. Higher health-related internet use scores indicate more health-related information-seeking behavior using the internet.

The two key independent variables were health literacy and access to technology devices. Health literacy is comprised of the total score of the following three health literacy items from the Behavioral Risk Factor Surveillance System Questionnaire [33] developed by the US Centers for Disease Control and Prevention, and was used as the primary measure of health literacy in this study: (1) “How difficult is it for you to get advice or information about health or medical topics if you needed it?” (2) “How difficult is it for you to understand information that doctors, nurses, and other health professionals tell you?” and (3) “You can find written information about health on the internet, in newspapers and magazines, and in brochures in the doctor’s office and clinic; in general, how difficult is it for you to understand written health information?” Each health literacy item was measured by a 5-point Likert scale ranging from 1 (“not at all”) to 5 (“always”), and the Cronbach α of the three items was .74. Access to technology devices was measured by a categorical variable assessing the type(s) of devices the respondent had access to. Respondents chose one of the following options: “no device,” “computer or tablet PC at home,” “mobile phone,” “mobile phone and computer or tablet PC at home,” “smartphone,” or “smartphone and computer or tablet PC at home.”

In addition to the measures on health literacy and technology devices, the sociodemographic and health information of participants, including gender, age, marital status (never married vs married/partnered), educational level (less than a bachelor’s degree, bachelor’s degree, or graduate degree and higher), annual health checkup in the past 12 months, any family cancer history, health status (poor/fair vs good/very good/excellent), and participation in a social group, were included as covariates.

### Data Analysis

We first investigated the association of health-related information-seeking internet use with sociodemographic characteristics using *t* tests for binary variables and *F* tests for categorical variables with more than two values. We report the Pearson correlation coefficients (*r*) for continuous variables. In particular, we focused on gender differences in health-related information-seeking internet use because previous studies have shown gender differences in internet use and health information–seeking behavior [13,15]. Next, we used multiple regression analyses for both binary and continuous outcome values of health-related internet use. We used logistic regression analysis for binary outcomes and ordinary least-squares regression analysis for the continuous health-related internet use score. We used heteroscedasticity robust standard errors for the multiple regression analyses. We conducted all analyses in Stata 14.1, using a 5% statistical significance level criterion.

## Results

### Sociodemographic Characteristics and Bivariate Analysis

Table 1 summarizes the sociodemographic characteristics of the study sample. A total sample of 614 was used in the study, with a majority of women. The mean health-related information-seeking internet use score was significantly higher for women than for men. The mean age of the sample was 41.87 years (SD 16.83), and the correlation between age and health-related internet use score was negligible (*r*=0.025, *P*=.54). Only 408 of the 614 participants (66.4%) in the sample reported their race/ethnicity.

The total health-related internet use score was significantly different among educational level groups, which was the lowest among individuals with less than a bachelor’s degree and was the highest among individuals with a graduate degree. This demonstrates that participants with higher education had higher health-related information-seeking internet use. The majority of respondents indicated having gone for an annual health checkup, and their health-related internet use score was significantly higher than that for those who had not had an annual health checkup. The majority of the sample reported that their health status was good, very good, or excellent; participants with a lower self-reported health status had higher health-related internet use scores than those with a higher self-reported health status.

The majority of the sample had a smartphone and a computer or tablet PC at home, followed by those with a smartphone only. The health-related internet use scores across the different technology device possession groups were significantly different, with the highest scores for those using a smartphone and a computer or tablet PC, followed by the scores for those who possessed a smartphone only. The mean score for those who owned a mobile phone and computer or tablet PC at home was higher than that of respondents who owned a computer or tablet PC only at home or those who had only a mobile phone. The score for those who did not own any technology device was the lowest, as expected. The mean value of the health literacy total score was 12.37 (SD 2.41, range 0-15), and the Pearson correlation coefficient with the health-related internet use score was 0.079, which is moderate (*P*=.04).

**Table 1:** Sociodemographic characteristics and their relation to health-related internet use of the study sample (N=614).

Characteristics^a^		Value	Health-related internet use^b^		
			Mean (SD)	Test statistic^c^	*P* value
**Gender, n (%)**				*t*_489.585_=2.95	.003
	Male	241 (39.3)	3.27 (2.49)		
	Female	373 (60.7)	3.87 (2.35)		
**Marital status, n (%)**				*t*_610.169_=1.41	.16
	Never married or other	315 (51.3)	3.50 (2.54)		
	Married or partnered	299 (48.7)	3.78 (2.28)		
**Educational level, n (%)**				*F*_2,611_=14.28	<.001
	Less than bachelor’s degree	197 (32.1)	3.06 (2.50)		
	Bachelor’s degree	262 (42.7)	3.60 (2.31)		
	Graduate degree	155 (25.2)	4.42 (2.30)		
**Annual health checkup, n (%)**				*t*_277.588_=4.31	<.001
	No	147 (23.9)	2.95 (2.14)		
	Yes	467 (76.1)	3.85 (2.46)		
**Family cancer history, n (%)**				*t*_200.472_=1.70	.09
	No	134 (21.8)	3.31 (2.57)		
	Yes	480 (78.2)	3.73 (2.37)		
**Self-reported health status, n (%)**				*t*_135.037_=2.81	.006
	Very poor/poor/fair	106 (17.3)	4.32 (2.85)		
	Good/very good /excellent	508 (82.7)	3.49 (2.30)		
**Participating in a social group, n (%)**				*t*_611.881_=1.81	.07
	No	321 (52.3)	3.47 (2.53)		
	Yes	293 (47.7)	3.82 (2.28)		
**Technology devices, n (%)**				*F*_5608_=6.28	<.001
	No device	10 (1.6)	0.80 (1.23)		
	Computer or tablet PC	7 (1.1)	2.57 (2.07)		
	Mobile phone	9 (1.5)	2.00 (1.80)		
	Mobile phone + computer or tablet PC	35 (5.7)	2.86 (2.05)		
	Smartphone	68 (11.1)	3.13 (2.06)		
	Smartphone + computer or tablet PC	485 (79.0)	3.87 (2.46)		

^a^The total sample size of each variable may not be the same as the total sample size of the study due to missing values.

^b^Based on health-related internet use total score (range 0-12).

^c^Two-tailed *t* test assuming unequal variances with Satterthwaite degrees of freedom for binary variables, and *F* test for categorical variables with more than two values.

Table 2 shows the results of the descriptive analysis on internet use for health information and health-related information-seeking internet use items. For the question asking about internet use for health information, 89.4% (549/614) of the sample reported that they have used the internet to look for health or medical information for themselves, with a significant gender difference. Among the 12 health-related internet use items, 6 items were significantly different between men and women at the 5% significance level; women used the internet more for these 6 items, which included “used email or the internet to communicate with a doctor or doctor’s office”; “used a website to help you with your diet, weight, or physical activity”; “looked for a health care provider”; “visited a social networking site such as Facebook or LinkedIn to read and share about medical topics”; “kept track of personal health information such as care received, test results, or upcoming medical appointments”; and “looked for health or medical information for someone else.”

**Table 2:** Descriptive analysis on health-related internet use.

Question		Total (N=614), mean (SD)	Males (n=241), mean (SD)	Females (n=373), mean (SD)	*t* statistic (df)^a^	*P* value
**Internet use for health information (Yes=1, No=0)**						
	In the past 12 months, have you used the internet to look for health or medical information for yourself? (N=614)	0.89 (0.30)	0.83 (0.37)	0.93 (0.25)	3.63 (379.481)	<.001
**Health-related internet use items^b^ (Yes=1, No=0)**						
	Looked for information about quitting smoking (N=610)	0.05 (0.21)	0.06 (0.23)	0.04 (0.19)	0.97 (447.531)	.33
	Bought medicine or vitamins online (N=610)	0.18 (0.38)	0.18 (0.39)	0.18 (0.39)	0.11 (504.817)	.91
	Participated in an online support group for people with a similar health or medical issue (N=609)	0.06 (0.24)	0.054 (0.23)	0.06 (0.25)	0.65 (540.24)	.52
	Used email or the internet to communicate with a doctor or doctor’s office (N=611)	0.46 (0.50)	0.39 (0.49)	0.51 (0.50)	2.91 (516.579)	.004
	Used a website to help you with your diet, weight, or physical activity (N=611)	0.52 (0.49)	0.47 (0.50)	0.55 (0.49)	2.02 (505.537)	.04
	Looked for a health care provider (N=608)	0.42 (0.49)	0.36 (0.48)	0.47 (0.50)	2.75 (519.359)	.006
	Downloaded health-related information to a mobile device such as an MP3 player, cell phone, tablet computer, or electronic book device (eg, download mobile apps) (N=610)	0.28 (0.45)	0.30 (0.46)	0.27 (0.45)	0.64 (495.534)	.52
	Visited a social networking site such as Facebook or LinkedIn to read and share about medical topics (N=611)	0.29 (0.45)	0.24 (0.43)	0.32 (0.47)	2.23 (537.405)	.03
	Wrote in an online diary or “blog” (ie, web log) about any type of health topic (N=605)	0.04 (0.19)	0.03 (0.18)	0.04 (0.20)	0.58 (538.839)	.56
	Kept track of personal health information such as care received, test results, or upcoming medical appointments (N=606)	0.43 (0.50)	0.37 (0.48)	0.47 (0.50)	2.59 (518.015)	.01
	Looked for health or medical information for someone else (N=599)	0.52 (0.49)	0.44 (0.49)	0.57 (0.49)	3.23 (497.276)	.001
	Done anything else health-related on the internet (N=610)	0.39 (0.48)	0.40 (0.49)	0.38 (0.49)	0.60 (506.309)	.55

^a^Two-tailed *t* test assuming unequal variances with Satterthwaite degrees of freedom.

^b^Cronbach α=.708.

### Multiple Regression Analyses

Table 3 shows the results of the logistic regression analysis that examined the association of health-related information-seeking internet use with health literacy, sociodemographic factors, and other health-related factors. Women were more likely to use the internet for health-related information-seeking, controlling for other factors, consistent with the bivariate gender comparison for the health-related internet use score (Table 1). Participants who had a postgraduate degree were more likely to use the internet for health-related information-seeking than those who had less than a bachelor’s degree. Respondents who had participated in a social group were more likely to use the internet for health information than those who were not in a social group. Respondents with a higher health literacy score were more likely to use the internet for health information. Compared with those who did not have a technology device, those who had a mobile phone and computer or tablet PC at home, those who had a smartphone only, and those who owned a smartphone and computer or tablet PC at home were more likely to use the internet for health-related information. The Wald χ^2^ test (Wald χ^2^_17_=53.30, *P*<.001) and pseudo *R^2^* (0.15) indicated a good model fit to the data.

**Table 3:** Multiple regression analyses for factors associated with health-related internet use for binary and continuous outcomes (N=614).^a^

Variable		Internet use for health information^b^		Health-related internet use total score^c^	
		Odds ratio (95% CI)	*P* value	Regression coefficient (SE)	*P* value
Female (Reference: Male)		2.68 (1.43-5.00)	.002	0.15 (0.05)	.007
Age		1.40 (0.95-2.07)	.09	0.11 (0.03)	<.001
Age^2^		0.99 (0.98-1.00)	.14	–0.002 (0.00)	<.001
Age^3^		1.00 (1.00-1.00)	.22	0.00001 (0.00)	.002
Married or partnered (Reference: Not married or partnered)		0.84 (0.41-1.71)	.63	–0.04 (0.05)	.46
**Education level**					
	Less than bachelor’s degree	Reference	N/A^d^	Reference	N/A
	Bachelor’s degree	1.19 (0.61-2.34)	.61	0.08 (0.06)	.18
	Graduate degree	2.99 (1.15-7.73)	.02	0.28 (0.07)	<.001
Annual health checkup (Reference: No annual health checkup)		1.19 (0.61-2.32)	.61	0.147 (0.06)	.02
Family cancer history (Reference: No family cancer history)		1.19 (0.63-2.27)	.59	0.07 (0.06)	.21
Good/very good/excellent health (Reference: Very bad/bad/fair)		0.61 (0.27-1.40)	.24	–0.22 (0.06)	<.001
Participating in a social group (Reference: not participating)		1.90 (1.03-3.52)	.04	0.13 (0.05)	.008
Log of health literacy total score		7.19 (2.07-25.02)	.002	0.35 (0.12)	.004
**Technology devices**					
	No device	Reference	N/A	Reference	N/A
	Computer or tablet PC	4.28 (0.27-68.49)	.31	0.43 (0.31)	.17
	Mobile phone	1.89 (0.16-21.81)	.61	0.42 (0.30)	.17
	Mobile phone + computer or tablet PC	13.37 (1.53-117.09)	.02	0.60 (0.19)	.002
	Smartphone	9.77 (1.48-64.61)	.02	0.73 (0.18)	<.001
	Smartphone + computer or tablet PC	8.13 (1.49-44.44)	.02	0.81 (0.17)	<.001

^a^Heterogeneity robust standard errors are used.

^b^Logistic regression for the dichotomous health-related internet use variable.

^c^Ordinary least-squares regression for the continuous health-related internet use total score; the natural logarithm of the health-related internet use score was used as the dependent variable.

^d^N/A: not applicable.

Table 3 also presents the results from the multiple ordinary least-squares regression analysis that investigated the association of the total health-related internet use score with health literacy, health-related factors, and sociodemographic factors. The health-related internet use score and the health literacy score were transformed using the natural logarithm function, which allowed us to interpret the results in approximate percentage changes in the analysis. Women were also more likely (by 14.50%) to have a higher health-related internet use score than men. Age was not significantly associated with the health-related internet use score when we added the age term only; however, age was significantly associated (at a 1% threshold) with the health-related internet use score when we included quadratic and cubic terms of age in addition to the linear term. This means that age was significantly associated with the health-related internet use score in a cubic manner, whereas there was no linear relationship between age and the health-related internet use score. Solving the cubic equation, we found that the health-related internet use score increased with age until about 38 years, decreased between the ages of 38 and 71 years, and increased again after the age of 71 years.

Respondents who had a postgraduate degree had a nearly 27.5% higher health-related internet use score than those who had less than a bachelor’s degree. Participants who had gone for an annual health checkup in the last 12 months had a nearly 14.7% higher health-related internet use score on average compared with those who had not gone for an annual health checkup. People who reported that their general health status was poor or fair had about a 22.6% higher health-related internet use score than those who reported their health status as good, very good, or excellent. The respondents who had participated in a social group had a 12.5% higher health-related internet use score than that of those who do not participate in a social group. People with a 10% higher health literacy score had a 3.5% higher health-related internet use score on average. In addition, health-related internet use scores for people who owned a computer or tablet PC or a mobile phone only were not significantly different from the scores of those without any kind of technology device. By contrast, compared with those without any technology device, people who possessed a mobile phone and computer or tablet PC had a 60.0% higher health-related internet use score, those who owned a smartphone only had a 72.5% higher health-related internet use score, and those who had a smartphone and computer or tablet PC had an 81.5% higher health-related internet use score. The *F* statistic (*F*_17,596_=10.30, *P*<.001) and *R^2^* value (0.20) indicated a good model fit to the data.

## Discussion

### Principal Findings

This study found that better health literacy and greater access to technological devices were associated with higher levels of health-related information-seeking behavior online. We found that the higher the health literacy level and the higher the accessibility to technological devices (eg, access to a mobile phone and computer or tablet PC at home or access to a smartphone), the more likely the respondents were to use the internet to seek health-related information. We also found differences in online health-related information-seeking behavior by sociodemographic characteristics; being female, having a graduate degree, and reporting a poor/fair health status were associated with higher usage of the internet for seeking health-related information.

It is possible that individuals with higher health literacy are more comfortable seeking out health information, are more adept at knowing what to search for and how to find it, and are more comfortable interpreting the information that they access. Our findings about access to technological devices are logical; individuals with more advanced, faster potential for online connectivity are more likely to use those devices for a variety of information-seeking purposes compared with individuals who have more limited access to technology. However, these findings signal a concerning disparity in access to information. As all types of information, including health information, are increasingly delivered online, individuals without access to efficient, effective technological devices are at risk of being left further behind.

The findings about differences in health-related information-seeking by sociodemographic characteristics indicate potential areas of inequity. For example, individuals with graduate degrees were more likely than individuals without college degrees to use the internet for health information. Again, this finding is not necessarily surprising, given already known disparities in access to and use of technology by socioeconomic status, but it could heighten disparities in access to health information [34,35]. On many of the individual items measuring online health-related information-seeking behavior, and in the multivariate model predicting ever using the internet for health information (vs never), women were more likely than men to obtain health information online. This may be related to a broader trend of gender differences in health care utilization [36]; however, this also presents an opportunity to implement more strategies to make online health information appealing and useful for men [37].

Additionally, we detected differences by age in online health information-seeking; younger adults and older adults were more likely than middle-aged adults to seek out health information online. For people of all ages who do not access health information online, it is important to ensure that comparable information is easily accessible in other forms. For example, for clinics and hospitals moving toward online-only scheduling and online communication with providers, careful thought should be given to who might potentially be left out and what alternative forms of communication, scheduling, and information delivery can and should be offered. For other sources of online health information (eg, websites, social media, hospital websites), care should be taken to ensure that people of all ages have equitable access to high-quality information, and that people who do not access such information online have equitable access to other forms of information.

### Limitations

This study should be considered in light of its limitations. We relied on a cross-sectional design, which therefore limits the ability to determine causality between online behavior and health literacy. We also did not have a random sample, but rather relied on a sample of adults who attended the Minnesota State Fair and were willing to respond to a survey. Nevertheless, we collected a robust sample capable of detecting meaningful differences in online health-related information-seeking behavior. To the extent that we did not capture a fully representative sample, we are likely to understate the differences we identified. We also did not examine intersectional differences by sociodemographic characteristics (eg, the potential multiplicative impact of gender and age), which should be explored more fully in future research. Finally, although we were able to examine differences in a range of health-related information-seeking behaviors, we were not able to determine the intensity or quality of these online interactions. Not all health information delivered online is good, and more attention should be paid to ensuring high-quality content and to educating the public on how to filter good from bad information.

### Practice Implications

Health information and health care are increasingly being delivered online. Prior research has shown that nearly 4 out of every 5 Americans are currently using the internet for health-related purposes [10,11]. Nearly half of the adults in our sample reported using the internet to communicate with their health care provider; more than half used it to look for information about their diet, weight, or physical activity; and more than half used the internet to look for health-related information for someone else. Clearly, the internet plays a large and growing role in how Americans manage and learn about their own health and the health of their loved ones. Thus, the internet has the potential to improve access to health information [9], to reduce barriers in communicating with health professionals [38], and to offer assistance to caregivers [39]. However, our findings also show that the internet is neither universally accessible nor universally used for obtaining health information. More attention needs to be paid to improving access to technology and to offering alternative forms of health information and communication for those without it. This might include expanding access to broadband internet and cellular connectivity in rural areas that do not have it; ensuring that public spaces such as libraries have ample access to computers where people can go online, as well as privacy protections for sensitive health information, such as cubby walls or dividers; and providing ample health information through other free sources, including libraries, clinics, and community spaces.

Finally, our study did not measure the quality of the information that individuals receive or the interactions they have. As health care, along with many other sectors of society, increasingly moves online, providers and educators are faced with the enormous task of assisting the public in filtering good information from bad and in advocating for high-quality health information online. This process might start early, in schools working with children on online literacy, but should also expand to include people of all ages, including in workplaces, senior centers, and health care settings.

### Conclusion

In this study, we demonstrated differences in online health-related information-seeking behavior according to the degree of health literacy, access to technological devices, and sociodemographic characteristics. We found that individuals with better health literacy, more access to sophisticated technological devices, and more education are more likely to access health information on the internet. Although these findings may not be surprising, they should be concerning; access to information is an important predictor of behavior [40,41] and disparities in access to health information may exacerbate disparities in health and health care access. Research, policy, and programmatic attention should be paid to improving access to technology, to ensure access to alternative forms of health information for those who cannot or will not access information online, and to improve the quality of online health information.
